# *In Silico* Design of BACE1 Inhibitor for Alzheimer's Disease by Traditional Chinese Medicine

**DOI:** 10.1155/2014/741703

**Published:** 2014-05-08

**Authors:** Hung-Jin Huang, Cheng-Chun Lee, Calvin Yu-Chian Chen

**Affiliations:** ^1^Department of Chinese Pharmaceutical Sciences and Chinese Medicine Resources, College of Pharmacy, China Medical University, Taichung 40402, Taiwan; ^2^School of Medicine, College of Medicine, China Medical University, Taichung 40402, Taiwan; ^3^Department of Biomedical Informatics, Asia University, Taichung 41354, Taiwan

## Abstract

The *β*-site APP cleaving enzyme 1 (BACE1) is an important target for causing Alzheimer's disease (AD), due to the brain deposition peptide amyloid beta (A*β*) require cleavages of amyloid precursor protein (APP) by BACE1 and *γ*-secretase, but treatments of AD still have side effect in recent therapy. This study utilizes the world largest traditional Chinese medicine (TCM) database and database screening to provide potential BACE1 inhibited compound. Molecular dynamics (MD) simulation was carried out to observe the dynamics structure after ligand binding. We found that Triptofordin B1 has less toxicity than pyrimidine analogue, which has more potent binding affinity with BACE1. For trajectory analysis, all conformations are tending to be stable during 5000 ps simulation time. In dynamic protein validation, the residues of binding region are still stable after MD simulation. For snapshot comparison, we found that Triptofordin B1 could reduce the binding cavity; the results reveal that Triptofordin B1 could bind to BACE1 and better than control, which could be used as potential lead drug to design novel BACE1 inhibitor for AD therapy.

## 1. Introduction

Alzheimer's disease (AD) is a progressive neurological disease of the central nervous system (CNS) that affects aging patients in the world [1–3]; the causes of AD are not well understood; recent studies indicate that the progression is associated with plaques accumulation and tau protein in the form of neurofibrillary tangles in the cortical region of brain [4–6]. The amyloid hypothesis indicated that amyloid is the initial cause of AD disease contributing to plaques accumulation; one of AD hallmarks is an aggregation of amyloid *β* (A*β*) leading to deposition of *β*-amyloid in the brain [7]. In A*β* reducing approaches, numerous studies demonstrate that amyloid vaccine can remove the amyloid plaques from the brains of the mice and reverse cognitive impairment [8–11], but in human clinical trials, the immunotherapy has side effects during the process of treatment, including autoimmunity [12] and high incidence of meningoencephalitis [13]; clearance of A*β* deposition still has problems for developing AD therapy. Hence, we focus on disrupt formation of A*β* from amyloid precursor protein (APP), cleavage by enzymes for AD prevention. The *β*-secretase is also called BACE1 (*β*-site amyloid precursor protein cleaving enzyme 1), which is an important enzyme in development of AD pathology. BACE1 cleaves transmembrane APP between residues 671 and 672, and carboxy-terminal fragment of APP is cleaved by *γ*-secretase, facilitating intramembrane proteolysis by the presenilin 1 (PSEN1) and presenilin 2 (PSEN2) [14, 15]. Subsequently the small 4 kilodalton of amyloid-A*β*1-40 and A*β*1-42 is generated by sequential *β* and *γ*-secretase cleavage of APP. Hence, the BACE1 has been recognized as a drug target for curing AD in many studies [16–18].

In this study, in order to design potential lead drugs for BACE1 inhibitor from nature products, computer-aided drug design (CADD) was employed to this research [19, 20], which includes molecular simulation and web server calculation [21, 22]. The nature products from world largest TCM database (TCM Database@Taiwan) were used to investigate more safety drugs [23], because TCM has been wildly used in clinical therapy for thousand years and also used in developing potential illness therapies [24–26]. The approach of CADD combined with TCM database has been wildly used to design new drugs successfully in many cases, including type II diabetes [27], neurotropic pain [28], head and neck cancer [29], hypertension [30], influenza [31–34], inflammation [35], breast cancer [36, 37], HIV virus [38], neuroprotection [39, 40], insomnia [41], erectile dysfunction [42], stroke [43–45], and weight loss [46, 47]. For drug targets, CADD should depend on risk factors study [48–51] and theories [52] to identify direction of each research. Hence, we present small molecular from world largest TCM database to analyze potential nature products by docking study between protein and small TCM compounds. Because BACE1 inhibitors are considered blood brain barrier (BBB) permeability, we also utilize ADMET prediction to evaluate the screening results from docking studies. Besides, we also employed molecular dynamics (MD) simulations to construct dynamic structure of BACE1 with docked ligands, observing the conformation changes over all simulation times.

## 2. Materials and Methods

### 2.1. Small Molecules and Protein Structure Preparation

The total numbers of TCM compounds from TCM Database@Taiwan were 61,000, and we employed the TCM compounds to search potent ligand as BACE1 inhibitor by docking study. We further used ADMET prediction and Lipinski's rule of five [53, 54] to estimate drug-likeness of the TCM compounds from docking results; these rules make them a likely oral drug in the human body. For ADMET prediction, we based on BBB penetration, CYP2D6 inhibition, and hepatotoxicity to analyze all docked ligands. The crystal structure of BACE1 was taken from PDB database (PDB code: 4JPE) [55]; the missing atoms and loops were corrected by* Prepare Protein module* under Accelrys Discovery Studio 2.5.5.9350 (DS 2.5) [56]; residues of BACE1 were protonated in pH 7.4 condition. We also used PONDR-FIT [57] to evaluate unfolded regions on BACE1 sequence for structure validation.

### 2.2. Docking Study

The volume of BACE1 inhibitor (1M7) in crystal structure of BACE1 was defined as binding site for screening TCM compounds through protein-ligand interaction; different poses of TCM compound were generated by Monte-Carlo techniques; docking study was performed by LigandFit module within DS 2.5. We utilized CHARMm force field [58] to minimize the conformation of each ligand. The energy function is as follows:

(1) U(R)=∑bondsKb(b−b0)2+∑angleKθ(θ−θ0)2 +∑Urey-BradleyKUB(S−S0)2 +∑dihedralsKφ(1+cos⁡⁡(nφ−δ))+∑bondsKω(ω−ω0)2 +∑non-bonded  pairs{εijmin⁡[(Rijmin⁡rij)2−2(Rijmin⁡rij)6]+qiqj4πε0εrij}+∑residesUCMAP(φ,ψ).

Minimization of each docking pose executes 1000 steps of Steepest Descent with an RMS gradient tolerance of 3 and followed by conjugate gradient. The generated conformation of ligands was docked into the defined binding site of BACE1; the ligand poses were calculated by various scoring functions including -PLP1, -PLP2, and -PMF.

### 2.3. Molecular Dynamics Simulation

The molecular dynamic simulation was carried out by GROMACS 4.5.5 package [59] to simulate the dynamic structure of BACE1 with docked compounds. We utilize charmm27 force field for the simulation system [60]. The distance between the edge of box and protein was set to 1.2 nm. Each protein-ligand system was placed in cubic cell containing water molecular by TIP3P model. Nonbonded interactions include repulsion, dispersion, and Coulomb terms. The repulsion and dispersion terms involve Lennard-Jones interaction [61] and Buckingham potential [62]; the cut-off distance of define van der Waals (VDW) residues was set to 1.4 nm. Long-range electrostatic forces were performed using the PME method [63, 64].

The equation of Lennard-Jones interaction is as follows:

(2) $$\begin{array}{c} U\left(r\right)=4\varepsilon \left[{\left(\frac{\delta }{\gamma }\right)}^{12}-{\left(\frac{\delta }{\gamma }\right)}^{6}\right]. \end{array}$$

The Buckingham potential is defined as

(3) $$\begin{array}{c} V_{bh}\left(r_{ij}\right)=A_{ij}\exp\left(-B_{ij}r_{ij}\right)-\frac{C_{ij}}{R_{ij}^{6}}. \end{array}$$

Topology files and parameters of small compounds in protein-ligand complexes were generated for GROMACS simulation by SwissParam web server [65]. Bonds lengths were constrained by the linear constraint solver (LINCS) algorithm. Na^+^ and Cl^−^ ion were randomly replaced with water molecular to neutralize the simulation systems, and the concentration was set as 0.145 M in solvent system. The energy minimization was used to stabilize the solvent system by Steepest Descent algorithm with 5,000 steps, the follow by equilibration performed under position restraints to equilibrated water molecular in the protein for 1 ns under constant temperature dynamics (NVT type) conditions. In final step, production running for 5000 ps under constant pressure and temperature dynamics (NPT type); all of the temperature simulation system was under 310 K condition. MD conformations are sampled every 20 ps and all frames are analyzed under GROMACS 4.5.5.

## 3. Results and Discussion

### 3.1. Docking Results

**Figure 1:**
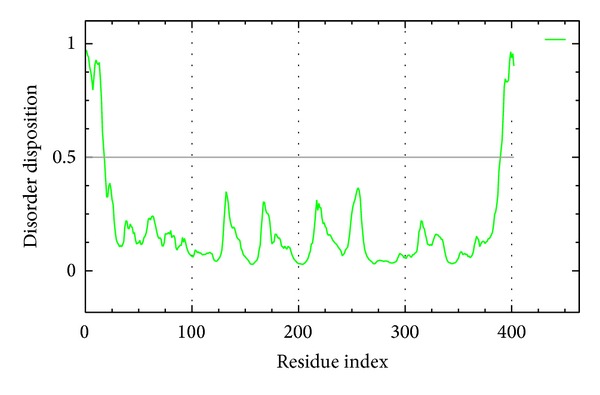
Disorder analysis of sequence of BACE1 from result of PONDR-FIT prediction; the value of disorder disposition above 0.5 indicate disorder residues.

**Figure 2:**
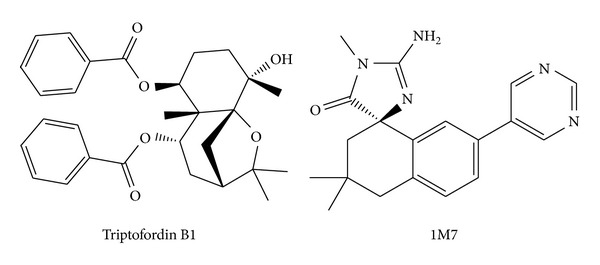
Chemical scaffolds of 1M7 (control) and Triptofordin B1.

**Figure 3:**
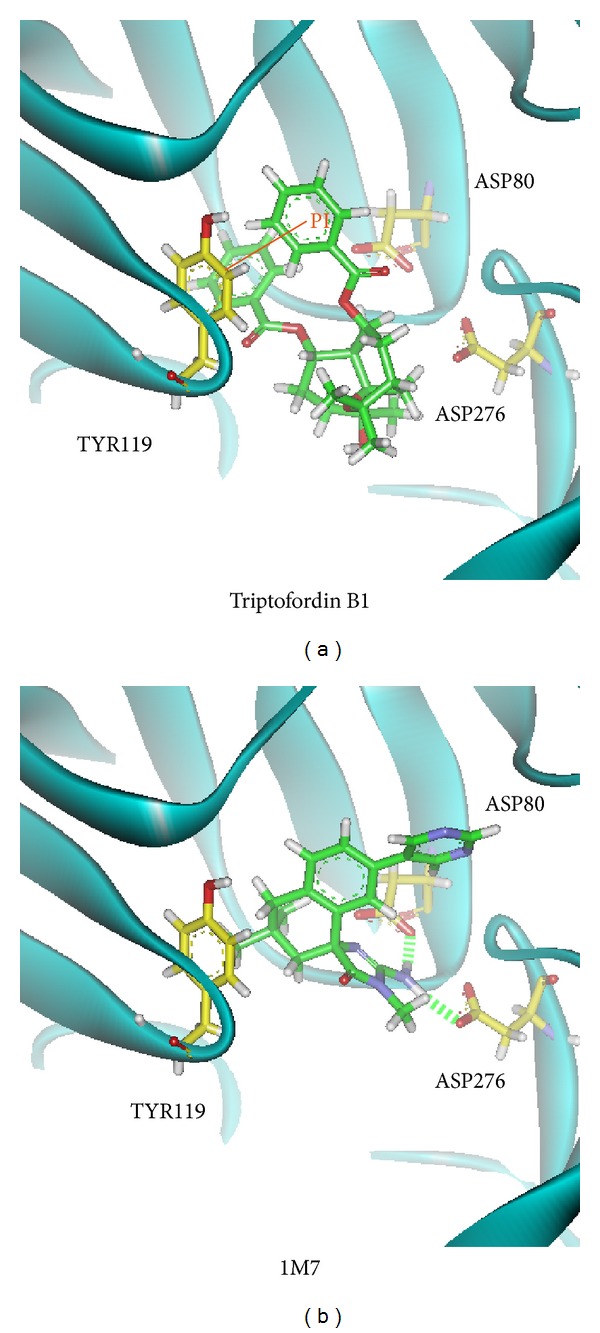
The docking poses of small compounds: (a) Triptofordin B1; (b) 1M7. Small compound and amino acids are colored in green and yellow, respectively.

We utilize PONDR-FIT [57] to understand the amino acids on binding region (GLN60, GLY61, ASP80, ILE158, ILE166, ASP276, GLY278, and THR279) of BACE1 are not disorder structure (Figure 1), and the values of disorder disposition are below 0.5, which indicate that the binding site of BACE1 is order structure, and the ligands binding may not affected by protein structure [66, 67]. For docking analysis, we based on -PLP1, -PLP2, and -PMF to evaluate the docking pose of traditional Chinese medicine (TCM) compounds. From scoring analysis, pyrimidine analogue R-50 (1M7) was regarded as control for comparison, which is synthesis BACE1 inhibitor from Hunt's study [55]. Top candidates with higher values of scores than 1M7 are shown in Table 1. For ADMET evaluation, all TCM candidates have no CYP2D6 inhibited and hepatotoxicity, suggesting that CYP2D6 may not be affected by these ligands in liver. The 1M7 has hepatotoxicity in ADMET analysis, indicating that our TCM candidates are safer than control. All docked ligands are ranked by -PMF score, due to the prediction of blood-brain barrier (BBB) penetration showing Diterpenoid EF-D with no penetration ability (BBB level = 4); the Triptofordin B1 has -PMF score (194.61) and medium penetration (BBB level = 2), which is better than 1M7 because of the low penetration (BBB level = 3) and low binding score (-PMF = 119.39). Triptofordin B1 is available in* Tripterygium wilfordii*; the herb extraction has therapeutic effect for SAMP8 mice with AD disease [68]. So we selected Triptofordin B1 for further studies; the chemical scaffolds of TCM candidates and 1M7 are shown in Figure 2. Docking pose of Triptofordin B1 displayed pi-pi interaction with TYR119; close residues include ASP80 and ASP276 (Figure 3(a)). 1M7 binding pose has H-bond with ASP80 and ASP276, but there is no pi interaction presented between residue and ligand. The data reveal that Triptofordin B1 has similar binding position with 1M7 and displayed stronger chemical interaction in BACE1 binding site. In further study, we utilized MD simulation to perform dynamic protein-ligand complexes for variation analysis.

### 3.2. Stability Analysis

**Figure 4:**
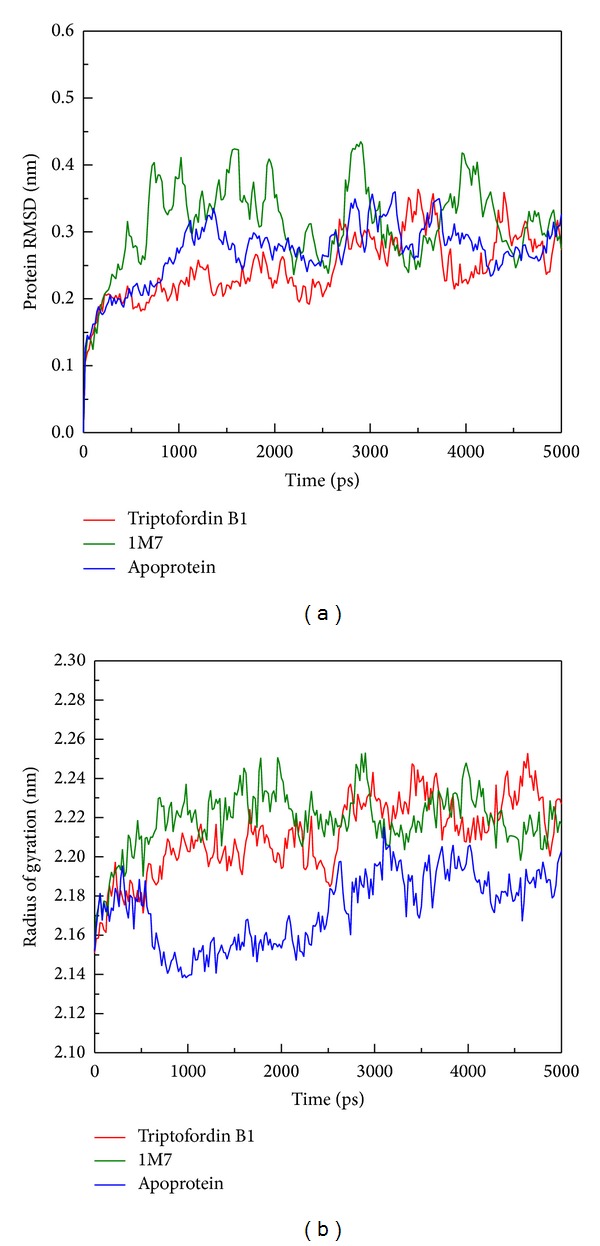
Plots of (a) protein RMSD and (b) radius of gyration from BACE1 during 5000 ps simulation time.

Structure of BACE1 with docked ligands includes Triptofordin B1 and 1M7 that were carried out by MD simulation, and we use protein structure of BACE1 with no ligand (Apoprotein) for comparison. The analysis result of protein root mean square deviation (RMSD) and radius of gyration (Rg) is shown in Figure 4. 1M7 displayed fluctuation from 500 to 4500 ps and was stable at 0.3 nm of protein RMSD. Triptofordin B1 and Apoprotein show similar trends; the protein RMSD remained stable in the region of 0.3 nm. The radius of gyration (Rg) analysis shows that the compactness of BACE1 with each ligand is less than the Apoprotein structure, because of the docked ligand combined with BACE1. From 3000 to 5000 ps of Rg analysis, the structure tends to be stable around 0.4 nm.

**Figure 5:**
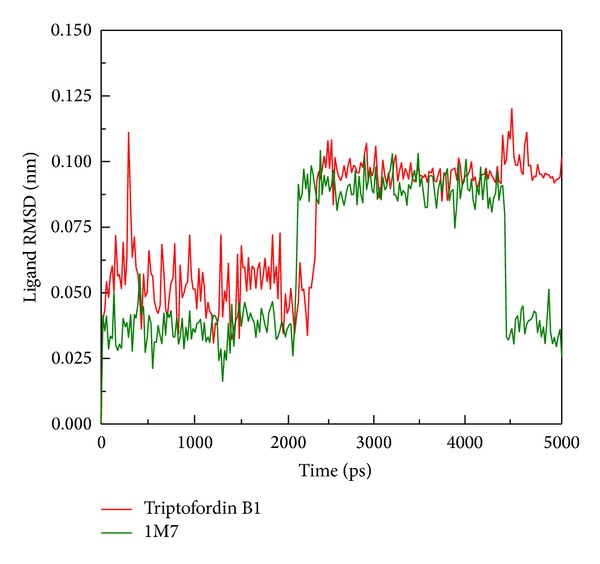
Plot of ligand RMSD values from BACE1 with docked ligands among 5000 ps simulation times.

**Figure 6:**
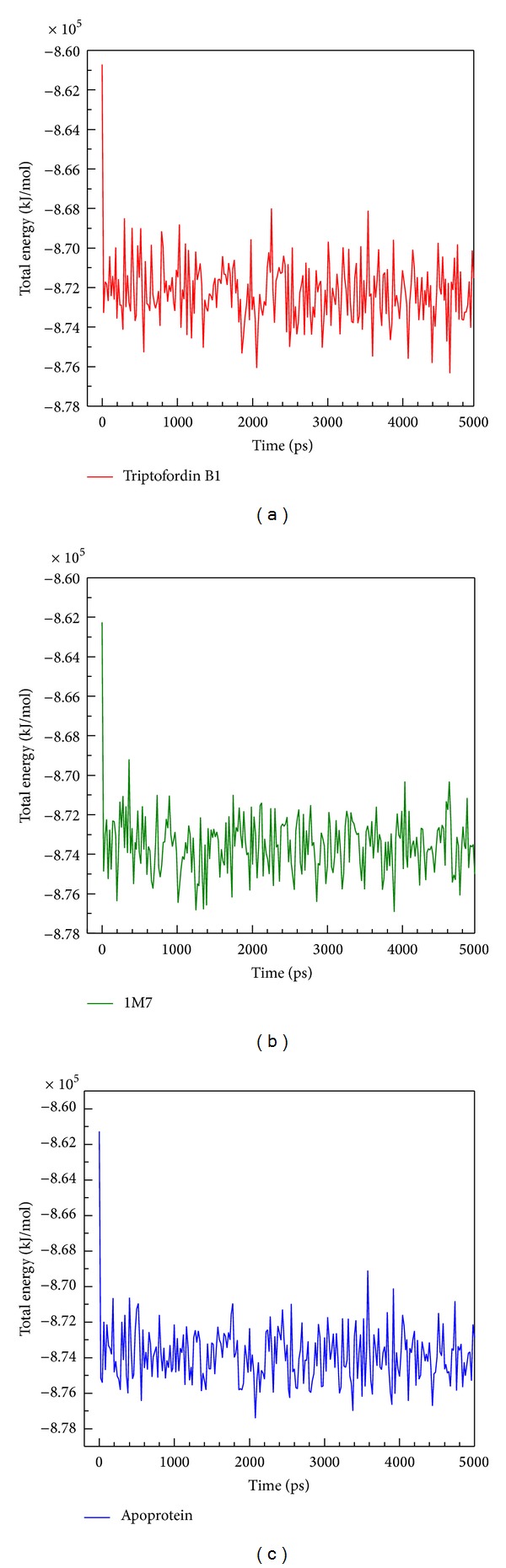
Total energy of BACE1 complexes: (a) Triptofordin B1; (b) 1M7; (c) Apoprotein among 5000 ps simulation times.

We further analyzed RMSD of each small molecular during MD simulation (Figure 5); ligand RMSD of Triptofordin B1 and 1M7 increases large fluctuation at 2000 ps; the value of ligand RMSD increased from 0.04 to 0.10 nm. Interestingly, 1M7 is decreased from 0.10 nm 0.04 nm after 4500 ps; this finding suggests that the region of 2000 to 4000 ps should be used to analyze the conformation of ligand binding. For total energy analysis, there significant increased values were observed at initial simulation time (Figure 6); the total energy is remained around −8.74 × 10^6^ kJ/mol for 1M7 and Apoprotein; the Triptofordin B1 was stable at −8.72 × 10^5^ kJ/mol. These results suggest that all structures of the complexes remained constant after initial simulation time; there is no significant fluctuation among all BACE1 structures.

### 3.3. Residues Fluctuation Analysis on the Binding Region

**Figure 7:**
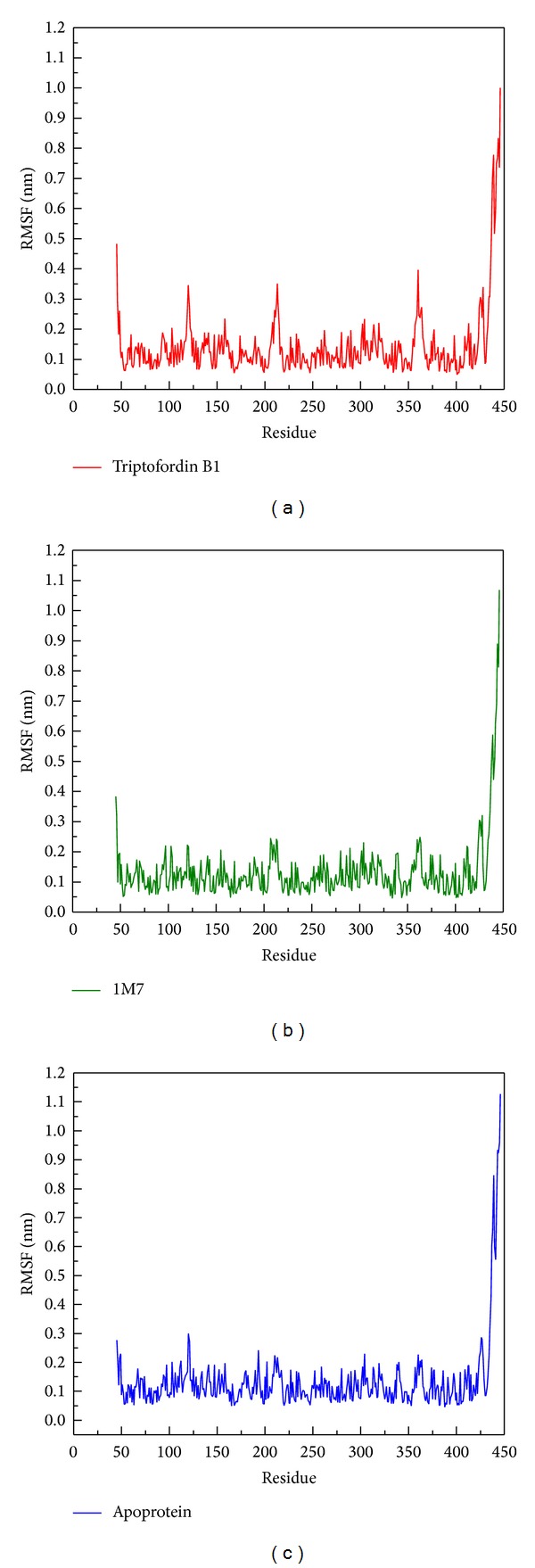
RMSF values of each residue of BACE1 with docked ligand: (a) Triptofordin B1; (b) 1M7 during 5000 ps simulation time.

We using root mean squared fluctuation (RMSF) to analyze the fluctuation of residues on protein binding site; the binding region (GLN60, GLY61, ASP80, ILE158, ILE166, ASP276, GLY278, and THR279) shows small flexibility (Figure 7). The largest fluctuation is observed from 425 to 450 residues, because these regions are far away from the binding site, indicating that the flexible amino acids do not affect protein-ligand interaction during MD simulation.

**Figure 8:**
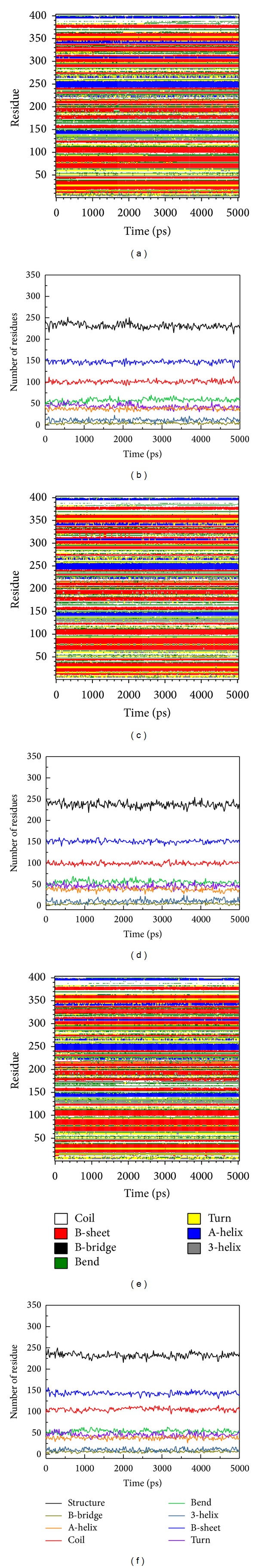
DSSP analysis of BACE1 complexes: (a) Triptofordin B1; (b) 1M7; (c) Apoprotein. Number of residues of secondary structure: (d) Triptofordin B1; (e) 1M7 (f) Apoprotein.

**Figure 9:**
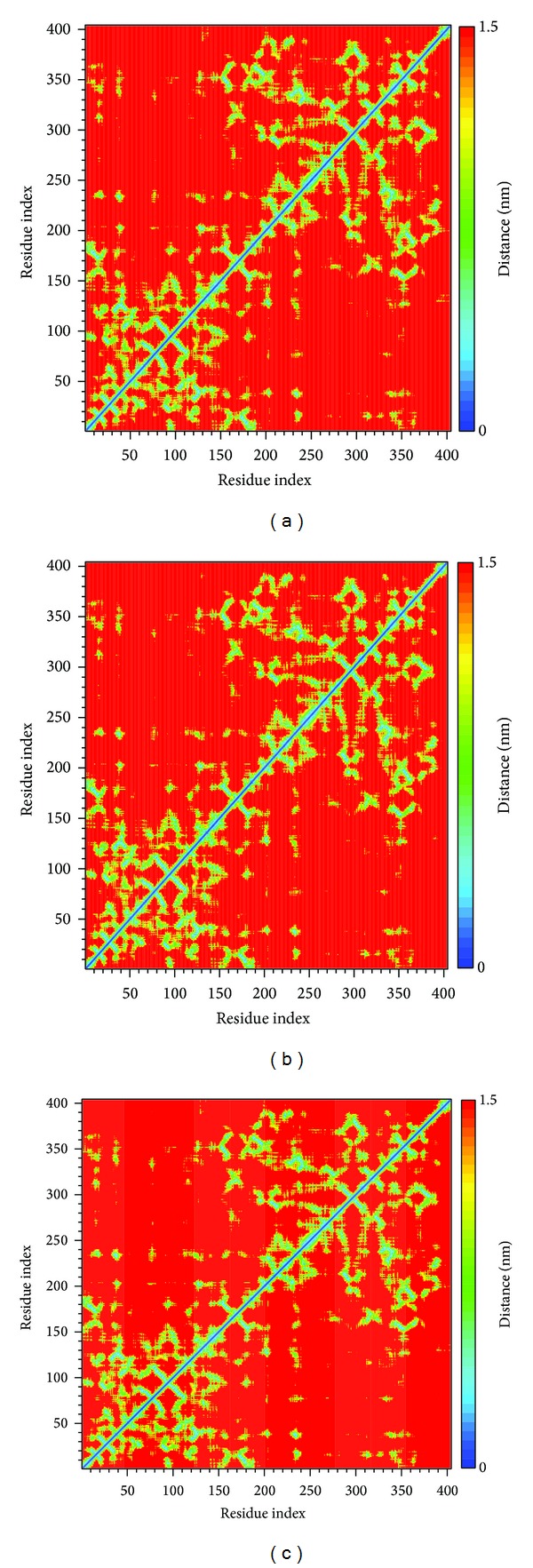
Matrix of smallest between each pair of amino acids in BACE1 complexes: (a) Triptofordin B1; (b) 1M7; (c) Apoprotein.

According to DSSP analysis, the number of helix and beta-sheet remained 150 and 100, respectively (Figure 8); the other secondary type also revealed no distinct changes. Besides, the distance for pair of each residue has no missing plot among all BACE1 structures during 5000 ps, (Figure 9). The results show that structure of BACE1 remained constant during all MD simulations.

### 3.4. Movement of Each Ligand Analysis

**Figure 10:**
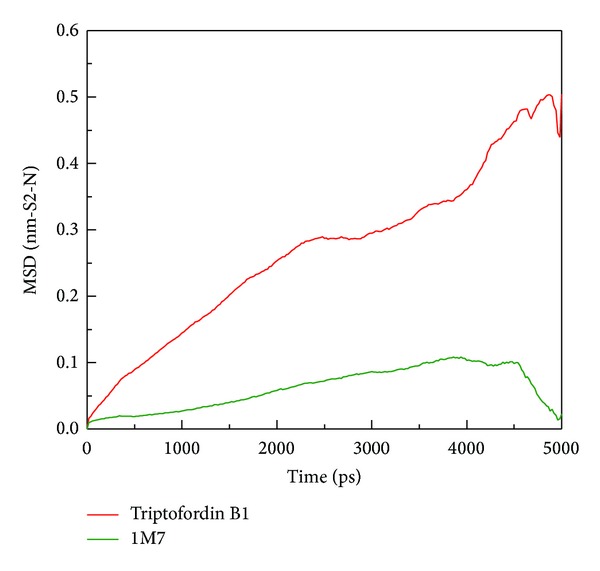
Mean square displacement (MSD) of different ligands during 5000 ps simulation times; the value of MSD indicates migration of ligands (Triptofordin B1 and 1M7) from initial site.

**Figure 11:**
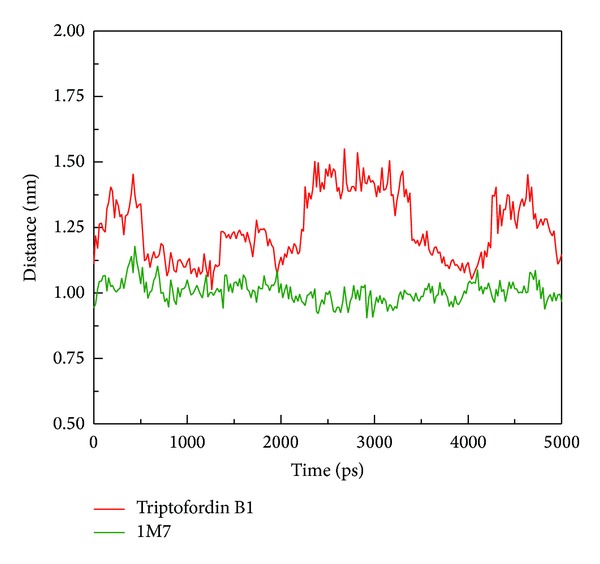
Distance between centers of mass of BACE1 and each ligand during 5000 ps simulation times.

The mobility of each ligand was analyzed by mean square displacement (MSD) (Figure 10); Triptofordin B1 increased MSD values to 0.3 nm at 2500 ps, and stabilizes until 4000 ps. 1M7 was stable below 0.1 nm and decreased MSD value at 4500 ps. In final simulation after 4500 ps, Triptofordin B1 further increased MSD values to 0.45 nm and tends to be stable to the end time. Here, we further analyze the distance between BACE1 and each ligand among 5000 ps (Figure 11). The distance between 1M7 and BACE1 displayed 1.00 nm before 2000 ps, but Triptofordin B1 increased to 1.50 nm from 2000 to 3500 ps, and the other wild increased distance occur from 4000 to 5000 ps. These results comparing with MSD analysis;the region of 200 to 3500 ps has significant change during dynamics simulation; in the next analysis, we focus on these regions of simulation time for further studies.

### 3.5. Clustering Analysis for Snapshot Observing

**Figure 12:**
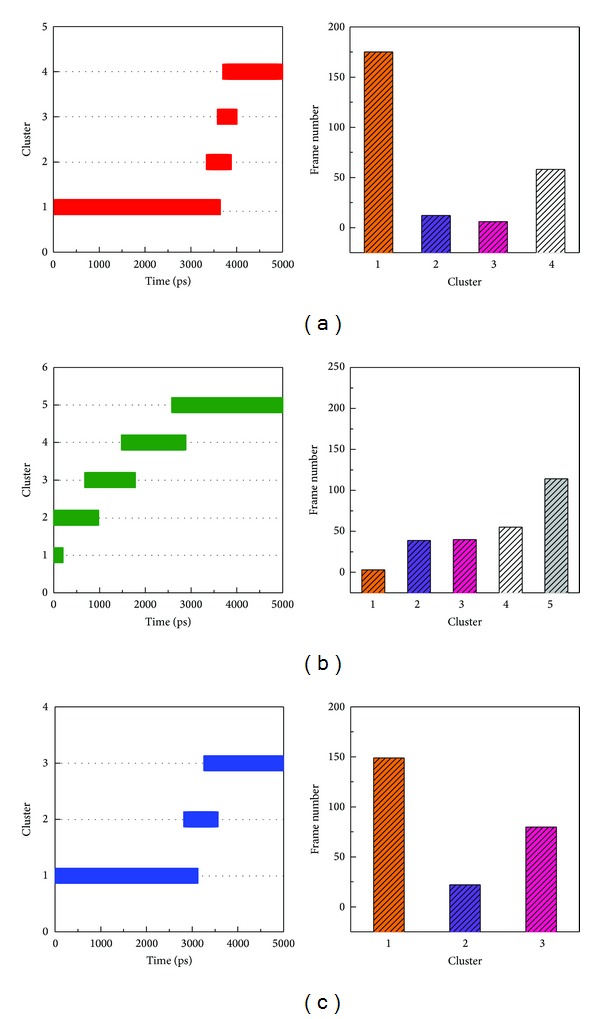
Clustering analyses among 5000 ps simulation times. (a) Triptofordin B1; (b) 1M7 (c) Apoprotein.

**Figure 13:**
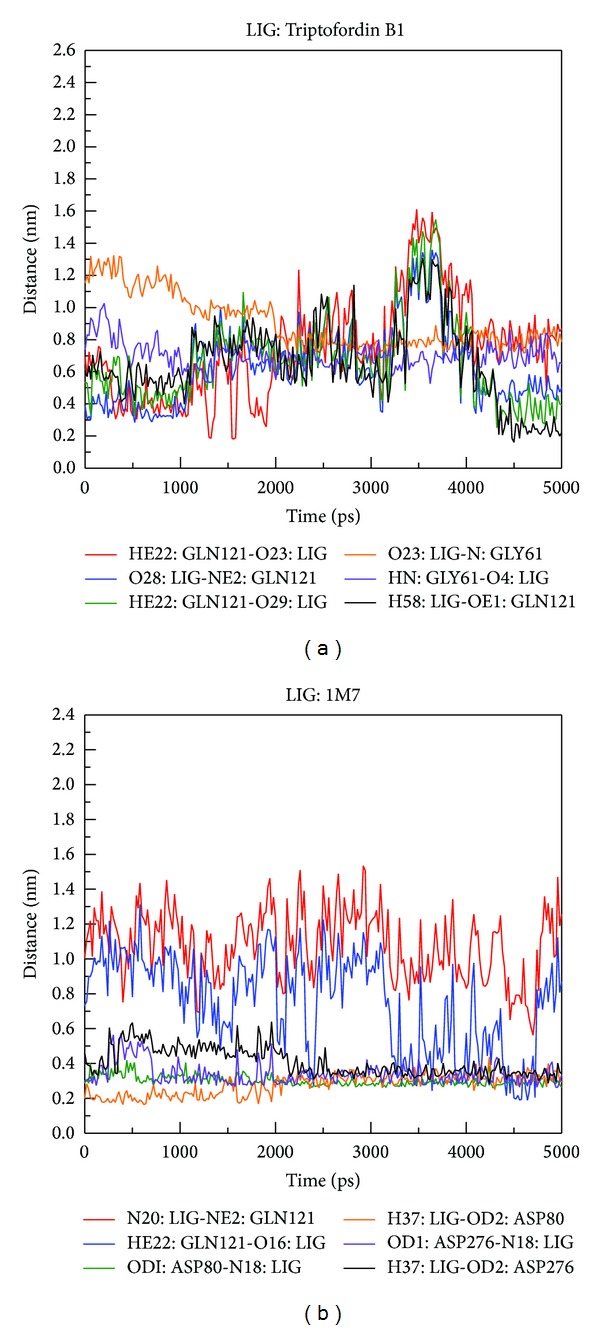
H-bond distance between residues atoms of BACE1 and ligands during 5000 ps simulation times: (a) Triptofordin B1; (b) 1M7.

**Figure 14:**
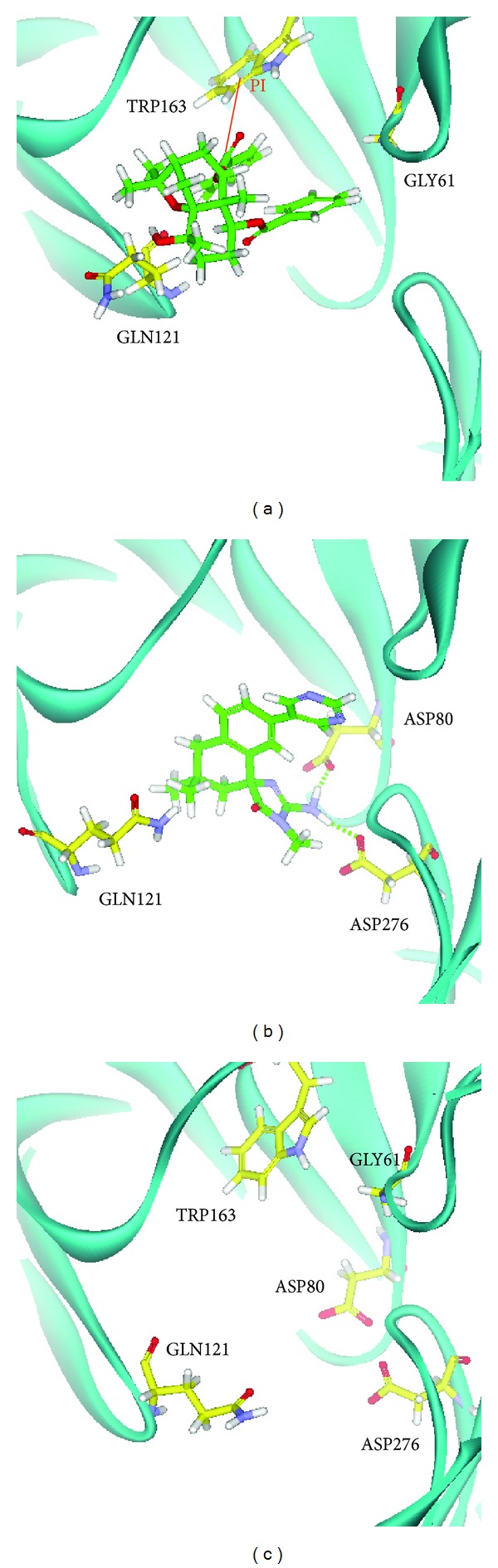
The middle structure from each final clustering group: (a) Triptofordin B1; (b) 1M7 (c) Apoprotein.

**Figure 15:**
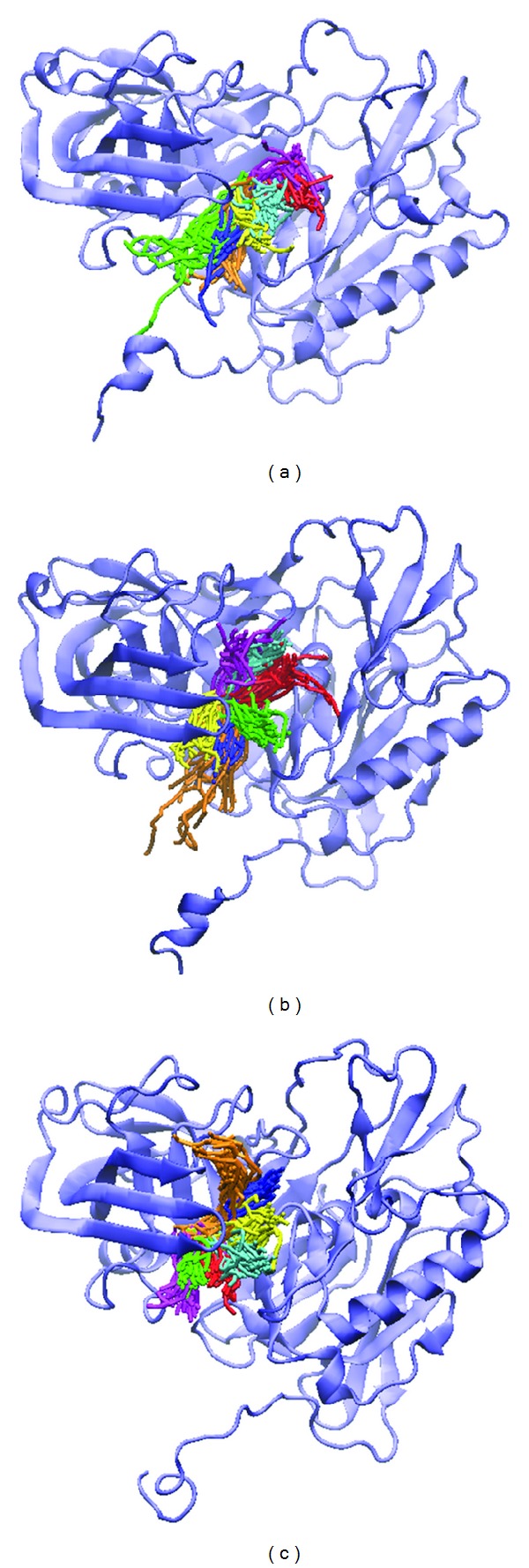
Predictions of ligand channel of (a) Triptofordin B1, (b) 1M7, and (c) Apoprotein by CAVER 3.0. The similar channels were collected in a group with same color.

In order to understand the most stable structure during the entire MD simulation for understanding the movement of BACE1, all frames of MD simulation were clustered into different subgroups (Figure 12); the similar MD conformations were grouped into the same cluster. For clustering results, each last group includes last 1000 ps (from 4000 to 5000 ps); hence, we selected the middle flams from each last group for further analysis from all MD complexes (Table 2). Before observing all snapshots from middle frames of last clustering group, we also calculate the distance of H-bonds for each ligand among all simulation times (Figure 13); GLN121 showed decreased distance after 4000 ps for Triptofordin B1 (Figure 13(a)). ASP80 and ASP276 remain revealed low distance with 1M7 (Figure 13(b)), suggesting that GLN121, ASP80, and ASP276 are essential amino acid for ligand binding. In snapshot analysis, we found that Triptofordin B1 could reduce the binding site, because the GLN121 has significant change, and presenting pi interaction with TRP163 (Figure 14), and in ligand channel analysis (Figure 15), we can see that the predicted channel of Triptofordin B1 is shorter than 1M7 and Apoprotein, suggesting that Triptofordin B1 could bind to BACE1 better than 1M7.

## 4. Conclusion

For ADMET analysis, Triptofordin B1 has more penetration than 1M7 and less toxicity, because 1M7 has hepatotoxicity in ADMET prediction. Three scoring functions, -PLP1, -PLP2, and –PMF, are higher than control. The structure of BACE1 analysis shows that the binding residues have less fluctuation after MD simulations, indicating the each ligand is not affected by protein residues. In migration analysis for Triptofordin B1 and 1M7, the stable region displayed from 3000 to 4000 ps; we utilize clustering analysis to observe this period simulation time. Triptofordin B1 could reduce the binding cavity of BACE1; the results reveal that Triptofordin B1 could bind to BACE1 and better than 1M7, which could be used as potential lead drug to design novel BACE1 inhibitor for AD therapy

## Figures and Tables

**Table 1:** Top ten candidates and control.

Name	-PLP1	-PLP2	-PMF	BBB Level	CYP2D6	Hepatotoxicity
Diterpenoid EF-D	79.04	75.67	195.32	4	0	0
*Triptofordin B1 *	*68.44 *	*62.28 *	*194.61 *	*2 *	*0 *	*0 *
Shionoside C	71.67	69.36	193.84	4	0	0
Jangomolide	72.27	67.01	187.36	3	0	0
Vibsanin W	77.98	76.62	184.31	4	0	0
2*α*,6*α*-Dihydroxybetulinic acid	59.52	57.72	183.93	2	0	0
Benzoylramanone	63.59	61.05	183.67	2	0	0
Pseurata D	63.28	62.87	180.42	4	0	0
Vibsanin I	78.18	72.43	179.15	4	0	0
1M7*	**70.70**	**52.10**	**119.39**	**3**	**0**	**1**

*Control.

^a^BBB level (blood brain barrier): high penetration = 1; medium penetration = 2; low penetration = 3; undefined penetration = 4.

^b^CYP2D6: noninhibitor = 0; Inhibitor = 1.

^c^Hepatotoxicity: Non-inhibitor = 0; inhibitor = 1.

**Table 2:** The middle conformation in each cluster from all MD conformations.

Cluster	Time of middle frame (ps)		
	Triptofordin B1	1M7	Apoprotein
1	1640	20	1680
2	3540	400	3180
3	3820	1380	4200
4	4300	2340	—
5	—	3760	—
